# The predictive power of neuropsychological measures in MCI: early detection of dementia conversion

**DOI:** 10.3389/fnagi.2026.1740033

**Published:** 2026-06-18

**Authors:** Concepcion Padilla, Sara E. Zsadanyi, Irina Aragón, Alejandra O. Morcillo-Nieto, Sara Rubio-Guerra, Nuole Zhu, José Enrique Arriola-Infante, Javier Arranz, Íñigo Rodriguez-Baz, Lucia Maure-Blesa, Jesús García Castro, Isabel Barroeta, María Carmona-Iragui, María Franquesa-Mullerat, Palak Mahant, Laura Videla, Laura Del Hoyo Soriano, Bessy Benejam, Daniel Alcolea, Alberto Lleó, Juan Fortea, Miguel Angel Santos-Santos, Ignacio Illán-Gala, Isabel Sala, María Belén Sánchez-Saudinós, Alexandre Bejanin

**Affiliations:** 1 Estudis de Ciències de la Salut, Universitat Oberta de Catalunya, Barcelona, Spain; 2 Sant Pau Memory Unit, IR SANT PAU, Hospital de la Santa Creu i Sant Pau, Barcelona, Spain; 3 Center of Biomedical Investigation Network for Neurodegenerative Diseases (CIBERNED), Madrid, Spain; 4 Department of Neurology, Torrecárdenas University Hospital, Almería, Spain

**Keywords:** cognitive domains, conversion, dementia, early detection, mild cognitive impairment, neuropsychological measures

## Abstract

**Background:**

With rising global life expectancy, early identification of dementia risk is crucial for implementing timely interventions. This study assesses the value of neuropsychological measures in stratifying the risk of mild cognitive impairment (MCI) progression over a 3 years period.

**Methods:**

This retrospective, longitudinal study included 349 MCI patients from the SPIN cohort who underwent an extended neuropsychological battery at baseline (2009–2021), enabling the calculation of composite cognitive domain scores; and follow-up clinical visits. A Ridge logistic regression following a data-driven selection of α within an Elastic Net tuning grid was utilized to identify key factors associated with dementia conversion.

**Results:**

Over 3 years, 37.8% of patients with MCI converted to dementia, 58.7% remained stable, and 3.4% reverted. At baseline, converters were significantly older, exhibited lower functional status, and demonstrated poorer performance across all cognitive domains compared to the stable and reverter groups. A regularized (Ridge) logistic regression, selected as the optimal model within an Elastic Net tuning grid; was used to identify the most robust predictors, yielding an Area Under the Curve (AUC) of 0.816 [95% CI: 0.769–0.862]. Predictor stability was assessed via 500 bootstrap iterations, which identified older age, male sex, higher education level, and lower baseline scores in MMSE, episodic memory, and executive functions as the most robust contributors to conversion. At the optimal probability threshold of 0.436 (Youden’s Index, *J* = 0.536), the model demonstrated robust discriminative power, specifically achieving a good specificity of 83.9% and a moderate sensitivity of 69.7% in forecasting progression to dementia.

**Conclusion:**

Findings underscore that baseline performance in episodic memory, and executive functions can effectively assist in screening for MCI progression. While the model shows good discriminatory potential, its clinical utility lies in risk stratification and identifying high-priority patients for monitoring, rather than serving as a definitive individual prognosis. Further research integrating longitudinal biomarkers is needed to refine these predictive frameworks and improve patient outcomes.

## Introduction

Mild Cognitive Impairment (MCI) is a condition characterized by a deficit in one or more cognitive domains that exceeds expectations for normal aging, yet does not significantly impair daily living activities (Petersen et al., 2009, 2018; Winblad et al., 2004). MCI is about four times more common than dementia and is highly prevalent among older adults, with higher rates in those with lower educational levels (Petersen et al., 2018). MCI has various causes, including systemic diseases, neurological conditions, medications, and mental health disorders (Ganguli et al., 2013; Petersen, 2016). Individuals with MCI are at a significantly higher risk of developing a neurodegenerative disease, including Alzheimer’s disease (AD), dementia with Lewy bodies, and Frontotemporal Dementia (Roberts and Knopman, 2013).

This diversity of etiologies explains the varied progression of the condition over time, reverting to normal cognition, remaining stable, or converting to dementia. The prevalence estimates differ depending on the definition of MCI, the clinical or population-based sample, or the age/sex distribution of participants (Ganguli et al., 2011; McGrattan et al., 2022). According to McGrattan et al. (2022), the annual conversion rate from MCI to dementia ranges between 10 and 15%, while reversion rates range between 4 and 15% in clinical settings and 29–55% in the general population (Ganguli et al., 2011). Tschanz et al. (2006) further indicated that 46% of individuals with MCI progressed to dementia after 3 years of follow-up in a population-based study.

Identification of MCI individuals at risk of converting to dementia is crucial for early intervention and care planning. Although major advancements in biological biomarkers have occurred over the past decade, access to these tools is still limited in many clinical settings, particularly in rural areas and low- and middle-income countries. Therefore, developing robust predictive models for MCI progression using clinical information (e.g., neuropsychological assessments, demographic factors) is essential to bridge this gap and ensure equitable diagnosis and intervention strategies globally. These models offer a comprehensive view of an individual’s cognitive status and support the development of personalized care strategies, which can significantly improve patient outcomes.

Evidence regarding the cognitive markers preceding dementia remains divided. Although specific memory deficits have been identified as primary predictors (Gainotti et al., 2014), a growing body of literature suggests that clinical conversion is more accurately captured by characterizing impairments across diverse cognitive functions (García-Herranz et al., 2016; Tabert et al., 2006). Demographic factors such as age, sex, and education level have also been associated with MCI outcomes. Hence, younger age, higher education levels, and greater cognitive reserve, together with preserved attention and executive functions and relatively intact memory functions, have been linked to a higher likelihood of MCI stability (Cooper et al., 2015; Petersen, 2016) or reversion to normal cognition (Roberts and Knopman, 2013). Yet, the predictive value of each cognitive domain and demographic data, when integrated into a single comprehensive model, is still not well established, especially in clinical cohorts, which better reflect the populations attending memory units.

Therefore, this study aims to overcome these limitations and investigate how neuropsychological measures and demographic factors can predict the 3-year progression of MCI in a large Spanish cohort from the Sant Pau Memory Unit, regardless of the information that biomarkers might add. Specifically, we relied on a 1 hour clinical neuropsychological screening battery to identify baseline differences in demographic data and cognitive profiles between MCI groups with different clinical trajectories (reverter, stable, and converter). We further evaluated the predictive capabilities of cognitive scores and demographic variables for MCI progression to dementia and developed an optimal predictive model.

## Materials and methods

### Study design and participants

In this retrospective, longitudinal study conducted at the Memory Unit of Hospital de la Santa Creu i Sant Pau, Barcelona, we analyzed data from MCI patients and cognitively unimpaired volunteers from the Sant Pau Initiative on Neurodegeneration (SPIN) cohort (Alcolea et al., 2019). The group of cognitively healthy older adults was included to (i) normalize participants’ cognitive performance relative to a reference group (i.e., W-score) and (ii) define baseline deficits within each MCI group.

All participants came to our facilities between 2009 and 2021. They underwent an annual clinical evaluation, except for those taking part in the study from 2020, who came biannually. This evaluation included comprehensive neurological history, physical and neurological examinations, structured caregiver interviews, and a neuropsychological assessment with both behavioral and cognitive evaluations.

Clinical diagnosis of MCI was performed between experienced neurologists and neuropsychologists from the Memory Unit, considering clinical history, functional performance, and psychometric scores, with clinicians blinded to any biomarker results. Individuals with MCI were included in this study if they were diagnosed as MCI by the multidisciplinary team. As such, they fulfilled the following criteria at baseline: (i) a cognitive global status of 3 on the Global Deterioration Scale (GDS Reisberg et al., 1982), (ii) impaired cognitive performance (defined as < 1.5 standard deviation below age- and education-adjusted normative scores) in two or more tests composing any of the cognitive domains, and (iii) a longitudinal evaluation with complete data. Cognitively unimpaired individuals were often spouses or children of patients who were informed about our studies at the outpatient clinics of the Sant Pau Memory Unit. By definition, they presented no deficits at the comprehensive cognitive assessment carried out at baseline and follow-up, and scored 1 on the GDS at both longitudinal evaluations. Exclusion criteria for the study included illiteracy, blindness, hearing impairment, patients on cognition-impairing medications, and a history of neurological or psychiatric diseases (e.g., psychosis), drug abuse, or systemic diseases that compromise longitudinal follow-up.

The study was approved by the Sant Pau Research Ethics Committee, following the standards for medical research in humans recommended by the Declaration of Helsinki. All participants gave written informed consent before enrolment. Confidentiality was guaranteed in accordance with current Spanish legislation (LOPD 3/2018).

### Neuropsychological evaluation

A 1-h neuropsychological assessment was administered to each patient by an experienced clinical neuropsychologist. To determine the global cognitive status, the Global Deterioration Scale (GDS, Reisberg et al., 1982) and Mini-Mental State Examination (MMSE, Folstein et al., 1975) were used. The GDS is a reliable tool for assessing the progression of primary degenerative dementia, with 7 stages: (1) No Cognitive Decline, (2) Subjective Cognitive Decline, (3) MCI, (4) Mild Dementia, (5) Moderate Dementia, (6) Moderately Severe Dementia, and (7) Severe Dementia. This scale is completed by an experienced clinician, typically a neurologist and a clinical neuropsychologist, based on a comprehensive clinical evaluation. The assessment integrates direct observations of the patient’s cognitive abilities, insights from a caregiver regarding daily functioning, memory complaints, behavioral changes, and a review of medical history, other cognitive test results, and relevant biomarkers.

Neuropsychiatric symptoms were evaluated using the Neuropsychiatric Inventory (NPI; Cummings et al., 1994) and Geriatric Depression Scale (DEPGDS; Yesavage and Sheikh, 1986). Functional disability was measured with the Interview for Deterioration in Daily Living in Dementia Scale (IDDD; Teunisse and Derix, 1997). Both the NPI and the IDDD are informant-based measures, meaning they were completed by a caregiver. For the IDDD, scores are interpreted such that higher values indicate a greater degree of functional disability (i.e., less overall functionality).

Specific cognitive tests were administered to assess the main cognitive domains: episodic and semantic memory, language, attention and executive functions, and visuospatial-visuoconstructive skills. *Episodic memory* was assessed using the Free and Cued Selective Reminding Test (FCSRT), the Consortium to Establish a Registry for Alzheimer’s Disease (CERAD) word list and figure, and The Rey–Osterrieth complex figure (ROCF). *Semantic memory and language* were measured using the 60-item Boston Naming Test (BNT), semantic fluency (1 min, animals), and the comprehension subtest from the AD Assessment Scale-Cognition (ADAS-Cog). *Attention and Executive functions* were evaluated with the Digits Forward and Backward from the Wechsler Memory Scale, phonemic fluency (1 min, “p”), and Trail-Making A and B (TMTA/B). Finally, *visuospatial and visuoconstructive abilities* were assessed with the figure copy of the ROCF and CERAD tests, the Number Location condition from the Visual Object Spatial Perception (VOSP) battery, and the superposition of figures from the Poppelreuter test for visual agnosia (Alcolea et al., 2019; Sala et al., 2008).

### Cognitive domain scores

To account for the influence of demographic factors on cognitive performance, all raw neuropsychological scores were converted to W-scores. W-scores provide a measure of the difference between an individual’s observed performance and their expected performance based on age, sex, and education-adjusted norms from a healthy control reference group. This approach allows for a more direct comparison of cognitive deficits across a heterogeneous cohort and reduces the need for demographic interaction terms within the regression model. To obtain composite scores for each cognitive domain, we first used the group of 105 cognitively normal individuals (46.5% male, mean age: 60 ± 11, mean years of education: 14 ± 4.6) to convert raw cognitive scores into W-scores (Bejanin et al., 2017; O’Brien and Dyck, 1995), corresponding to Z-scores adjusted for age, sex, and education. Specifically, we performed linear regressions in the healthy control group to estimate the effect of demographic variables (age, sex, and education) on cognitive performances. We then used the coefficients and residuals of these regressions to compute W-scores for each participant using the following formula:

W-score = [(patient’s raw value) - (patient’s expected value)]/standard deviation of the residuals in the healthy control group, where patient’s expected value corresponded to the predicted value in the healthy control group given cases’ age, sex and education. Subsequently, patient W-scores were averaged within each cognitive domain (for details on the weight for each cognitive score, see Supplementary Figure 1).

Some patients had missing data for specific cognitive tests (see Table 1 and Supplementary material). To maximize data retention while limiting bias due to excessive missingness, COMPADAS-15 (74.8% missing) and the Rey Test (>55% missing) were excluded a priori. For the remaining measures, composite scores were derived using available observations. The vast majority of these variables presented less than 5% missing data, with the specific exceptions of CERAD list recall/recognition and TMT-B (precise per-test missingness is provided in Supplementary Table S1).

**TABLE 1:** Demographic and clinical characteristics of MCI patients at baseline.

Baseline profile and clinical metrics	Reverter *N* = 12	Stable *N* = 205	Converter *N* = 132	*p*-value	*n*
Sex				0.13	349
Female	8 (67%)	115 (56%)	61 (46%)		
Male	4 (33%)	90 (44%)	71 (54%)		
Age (years)	62.10 (7.42)	70.15 (7.49)	72.89 (7.21)	**<0.001**	349
Education (years)	12.83 (4.22)	10.59 (4.82)	10.34 (4.64)	0.22	349
Follow-up time (years)	3.25 (0.52)	3.19 (0.57)	2.77 (0.75)	**<0.001**	349
MMSE	28.67 (1.15)	26.97 (2.25)	25.81 (2.42)	**<0.001**	347
DEPGDS	12.33 (7.10)	12.22 (6.25)	11.77 (6.92)	0.72	345
NPI	18.00 (16.67)	13.04 (23.18)	14.85 (19.97)	0.51	195
IDDD	36.29 (4.75)	37.78 (5.11)	40.48 (7.37)	**<0.001**	288
Diagnosis					
AD			32 (41%)		
FTLD			14 (18%)		
LBD			13 (17%)		
Others			10 (13%)		
Psychiatric			4 (5.1%)		
Vascular			5 (6.4%)		

Results are mean (standard deviation) for continuous variables or frequency (%) for categorical. In bold, *p* < 0.05. MMSE, Mini-Mental State Examination; DEPGDS, Geriatric Depression Scale; NPI, Neuropsychiatric Inventory; IDDD, Interview for Deterioration of Daily Life in Dementia. AD, Alzheimer’s Disease; FTLD, Frontotemporal Lobe Dementia; LBD, Lewy Body Dementia.

### Categorization of MCI based on evolution

Clinical status was established based on the GDS score at approximately 3 years of follow-up. This measure was directly available for 60.17% of participants enrolled prior to 2020, who underwent annual follow-ups. For participants enrolled from 2020 onwards, follow-up was conducted biennially; consequently, visit at 4 years (22.06%) was selected as the closest approximation to a 3-year time point. For the most recently enrolled participants, only 2-year follow-up data (17.77%) were available at the time of analysis.

Individuals with MCI were categorized into three groups based on their GDS scores at the follow-up visit: “reverters,” “stable,” and “converters.”

(1) The reverter group (*n* = 12) consisted of individuals who obtained a GDS score of less than 3 at the follow-up visit.

(2) The stable group (*n* = 205) consisted of patients who maintained a GDS score of 3 throughout the follow-up visit.

(3) The converter group included individuals with a GDS score greater than 3 at the follow-up visit.

### Statistical analysis

All statistical analyses were performed with the R software, version 4.4.3 (R Foundation for Statistical Computing). Between-group differences for baseline characteristics were analyzed using χ^2^ tests for categorical data and Kruskal-Wallis test in continuous variables. Pairwise group comparisons were performed using Wilcoxon rank-sum tests. To control for the inflation of Type I error rate due to multiple testing, *p*-values were adjusted using the Holm-Bonferroni correction. All statistical tests were two-tailed, and a *p* < 0.05 (after adjustment) was considered statistically significant.

Logistic regressions were conducted to identify the cognitive domains (i.e., episodic memory, semantic memory and language, attention and executive functions, and visuospatial functions), and demographic (i.e., age, sex, and education) variables at baseline that best predicted the conversion from MCI to dementia, i.e., MCI converters vs. MCI non-converters (including both stable and reverter MCI groups). Initially, univariable analyses were performed to assess the predictive value of each regressor. We also explored if any specific neuropsychological test exhibited higher predictive ability than others. Standardized regression coefficients were used to compare the strength of associations between variables and the outcome. Subsequently, a multivariable analysis incorporating all variables of interest was conducted.

A regularized (Ridge) logistic regression, embedded within an Elastic Net tuning grid, was implemented to assess the relative contribution of demographic factors and neuropsychological composites to the risk of dementia conversion within 3 years of MCI diagnosis. This approach enabled effective handling of multicollinearity among cognitive scores and mitigation of overfitting. All predictors were standardized before model fitting. We tuned the mixing parameter α over a grid search on the interval [0, 1] with increments of 0.1. The optimal α was selected based on the value that maximized the internal cross-validated Area Under the Curve (AUC). The optimal α was 0, indicating that a Ridge penalty provided the best cross-validated fit, suggesting that predictive information was distributed across the variable set rather than concentrated in a few sparse features. The penalty parameter λ was selected using 5-fold cross-validation, retaining λ_*min*_ (0.034), corresponding to the model with the minimum mean cross-validated error. To evaluate the stability of the estimates in the absence of intrinsic sparcity, we performed 500 bootstrap resamples of the dataset. Predictors were considered robust if their 95% bootstrap confidence intervals (CI) consistently excluded zero. The model’s reliability was further supported by maintaining an Events-per-Variable (EPV) ratio above the recommended threshold of 10 (Peduzzi et al., 1996). The sensitivity and specificity of the models were evaluated by calculating the AUC using Receiver Operating Characteristic (ROC) curves. To assess the internal validity of the integrated Ridge model and to account for potential overfitting, we performed a bootstrap-based internal validation (500 iterations) using the Harrell approach (via the rms package in R). We derived an optimism-corrected AUC by calculating Somers’ D_*xy*_ during the bootstrap procedure and converting it to the area under the curve [*AUC = (D_*xy*_ + 1)/2)*]. Finally, DeLong statistics were utilized to compare performance between models, with the threshold for statistical significance set at *p* < 0.05.

## Results

### Study population

This study included 349 MCI patients who came to the hospital for at least two visits at baseline and follow-up (for demographic data, see Table 1). The mean (SD) number of follow-up visits was 1.64 ( ± 0.67) over an average time interval of 3.1 years (ranging from 2.6 to 3.4 years). The mean follow-up duration for the stable group (3.2 ± 0.56 years) was significantly longer than that of the converter group (2.8 ± 0.75 years; *p* < 0.001). This indicates that stable patients remained cognitively stable despite having a longer observation window in which to potentially convert, reducing the likelihood that their group assignment was due to insufficient follow-up time. Twelve (3.4%) patients became reverters, 205 (58.7%) remained stable, and 132 (37.8%) converted to dementia after 3 years of follow-up. Of those who converted to dementia, a substantial percentage (41%) developed AD.

**FIGURE 1:**
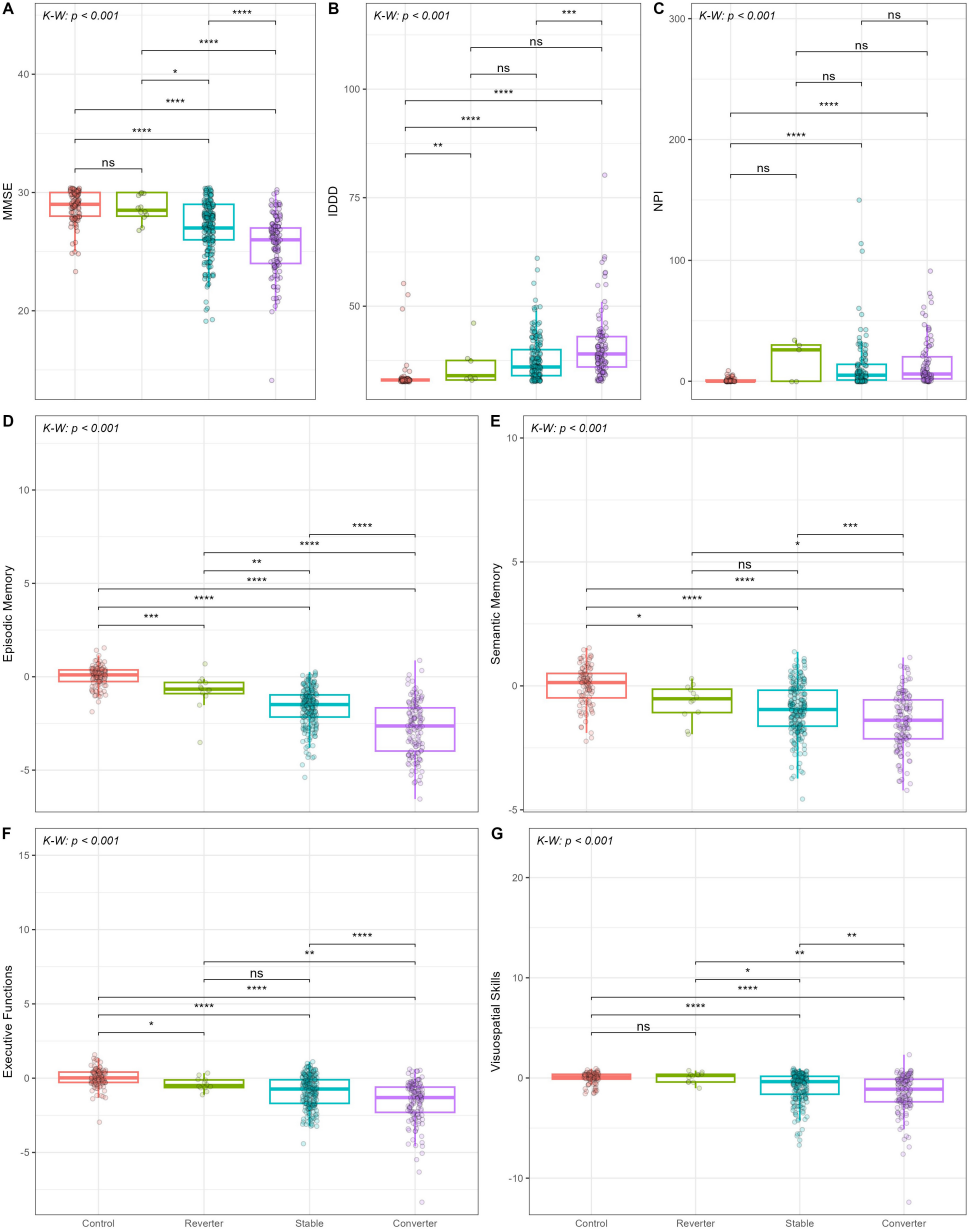
Behavioral and functional differences between controls and MCI groups. Boxplots showing the performance of the groups (controls, MCI reverters, MCI stable and MCI converter) on measures of **(A)** Mini-Mental State Examination (MMSE), **(B)** Interview for Deterioration in Daily Living in Dementia Scale (IDDD), **(C)** Neuropsychiatric Inventory (NPI), **(D)** Episodic Memory, **(E)** Semantic Memory, **(F)** Executive Functions, and **(G)** Visuospatial skills. The significance of the group effect was determined using the Kruskal-Wallis test. Pairwise group comparisons were performed using Wilcoxon rank-sum tests, with *p*-values adjusted for multiple comparisons using the Holm-Bonferroni method. Note: ns, no significant; *p < 0.05; **p < 0.01; ***p < 0.001, ****p < 0.0001 indicate statistical significance. K-W, Kruskal-Wallis; ns, no significance.

Controls obtained lower scores than MCI groups on the IDDD (all *p <* 0.01, Figure 1B). They also performed better than the stable and converter groups (*p <* 0.001), but not the reverters group in the MMSE (*p* = 0.45, Figure 1A) and NPI (*p* = 0.16, Figure 1C).

Comparisons between the three MCI groups did not reveal significant differences in sex, years of education, or baseline DEPGDS score (all *p* > 0.05, Table 1). However, there were significant age differences (*p <* 0.001, Table 1): the reverter group (62.10 ± 7.42) was the youngest, followed by the stable (70.15 ± 7.49) and then the converter (72.89 ± 7.21) groups. Additionally, they differed significantly on MMSE (all *p <* 0.001, Figure 1A and Table 1), with lower scores for the converter group (25.81 ± 2.42), followed by the stable (26.97 ± 2.25), and then the reverter (28.67 ± 1.15). Higher functional disability, as measured by the IDDD, was observed in the converter group relative to the stable group (*p* < 0.001; Figure 1B), while scores were comparable to those of the reverter group (*p* = 0.09). MCI groups did not differ in NPI scores, but each of them obtained higher NPI scores compared to the control group (*p* < 0.001), but not reverted group (*p* = 0.16, Figure 1C). Given the limited sample size of the reverter group (*n* = 12), statistical comparisons involving this subgroup should be interpreted with caution.

### Neuropsychological performances

The main effect of the group was significant (*p <* 0.001) for all cognitive domains. Post hoc test showed that each MCI group had significantly lower scores than controls on most measures: episodic memory (all *p <* 0.001, Figure 1D), semantic memory (all *p <* 0.05, Figure 1E) and executive functions (all *p <* 0.05, Figure 1F). For visuospatial skills, the controls showed better performances than the stable and converters (*p <* 0.001, Figure 1G), but not the reverters (*p* = 0.69; Figure 1G).

Among MCI groups, the converters had significantly lower scores than the stable and reverters in all cognitive measures: episodic memory (*p <* 0.001; Figure 1C), semantic memory (*p <* 0.001 and *p <* 0.05, respectively; Figure 1E), executive functions (*p <* 0.001 and *p* < 0.01, respectively; Figure 1F) and visuospatial skills (*p* = 0.01; Figure 1G). The stable group had a lower score than reverters in episodic memory (*p <* 0.01) and visuospatial skills (*p <* 0.05), but not in semantic memory (*p* = 0.28), and executive functions (*p* = 0.13, Figures 1D–G). Due to the small size of the reverter cohort (*n* = 12), findings regarding this group may lack sufficient statistical power and should be considered preliminary.

### Predictors of conversion—univariable models

**FIGURE 2:**
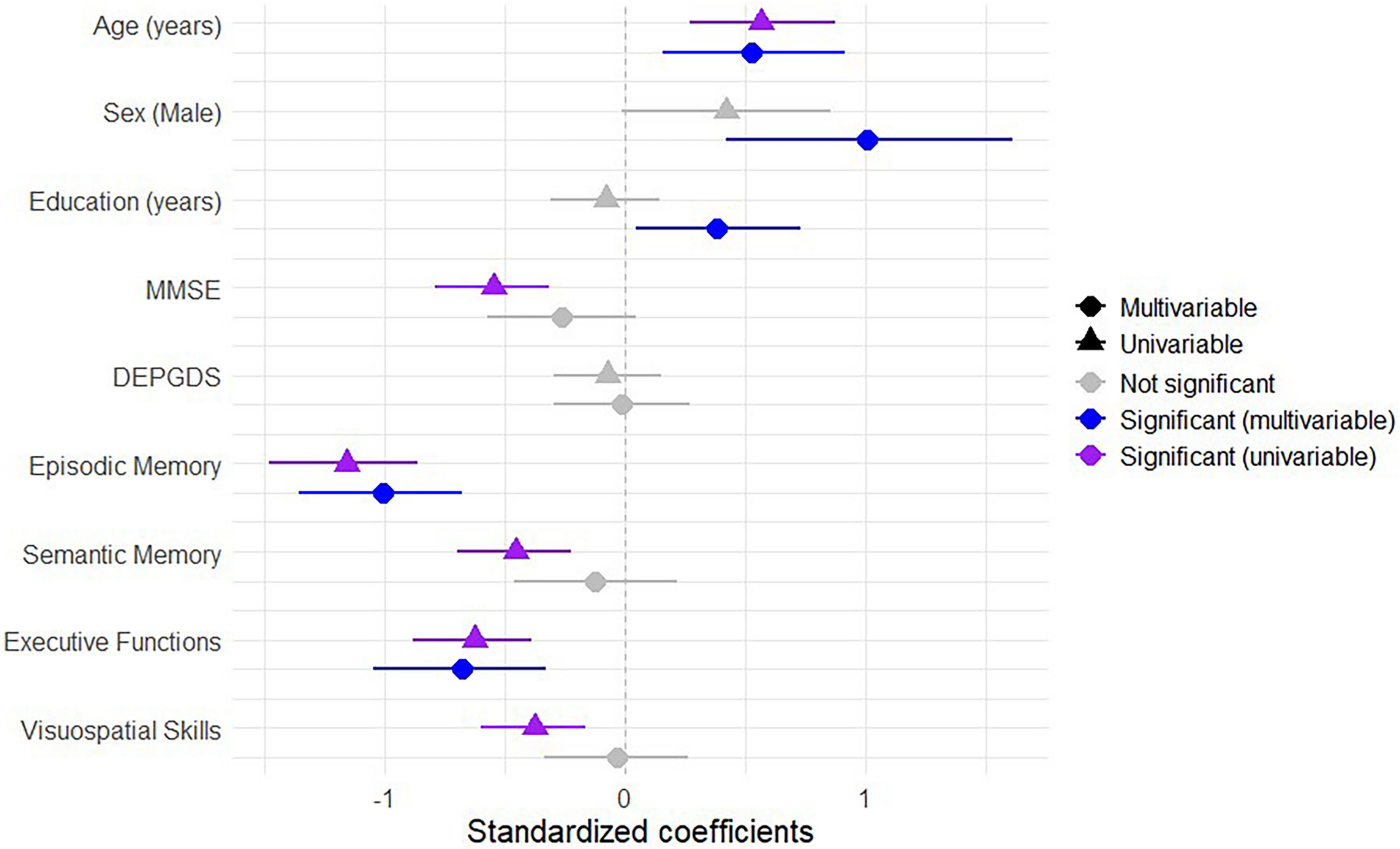
Univariable and multivariable logistic regression models for progression from MCI to dementia. The triangle and circle shapes represent the standardized coefficients for univariable and multivariable models, respectively. The lines represent the 95% confidence interval. Gray color indicates non-significant values, while blue and purple indicate significant values. MMSE, Mini-Mental State Examination; DEPGDS, Geriatric Depression Scale; EF, Executive Functions.

To evaluate the ability of cognitive and demographic variables to predict progression from MCI to dementia, we performed a binary logistic regression analysis (converter vs. non-converters). The univariable analysis (Figure 2), in which each variable was assessed individually, showed that older age acted as a significant risk factor, with each one-standard-deviation (SD) increase associated with a 76% increase in the odds of progression (*p <* 0.001, OR = 1.76, 95% CI: 1.31–2.40). Conversely, higher baseline scores across all cognitive domains demonstrated a protective effect. Episodic memory emerged as the most robust predictor, where every 1 SD increase in performance was associated with a 69% reduction in the risk of conversion (*p* < 0.001, OR = 0.31, 95% CI: 0.23–0.42). Similarly, higher performance in executive functions (*p* < 0.001, OR = 0.54, 95% CI: 0.41–0.68), MMSE (*p <* 0.001, OR = 0.58, 95% CI: 0.45–0.73), semantic memory (*p* < 0.001, OR = 0. 63, 95% CI: 0.50–0.80), and visuospatial skills (*p* < 0.001, OR = 0.69, 95% CI: 0.55–0.85) significantly lowered the odds of progression, with risk reductions ranging from 31 to 46% per SD increase. In contrast, sex, education, and depressive symptoms (DEPGDS) did not show significant predictive value in the univariable models (Figure 2 and Table 2).

**TABLE 2:** Logistic regression models for predicting conversion from MCI to dementia.

Predictor variable	Univariable OR [95% CI]	*p*-value	Multivariable OR [95% CI]	*p*-value
Age	1.76 [1.31–2.40]	**<0.001**	1.70 [1.17–2.50]	**0.006**
Sex (Male)	1.52 [0.99–2.36]	0.058	2.73 [1.53–5.01]	**<0.001**
Education	0.92 [0.73–1.16]	0.481	1.47 [1.05–2.08]	**0.027**
MMSE	0.58 [0.45–0.73]	**<0.001**	0.77 [0.56–1.05]	0.098
DEPGDS	0.93 [0.75–1.16]	0.531	0.99 [0.74–1.31]	0.932
Episodic memory	0.31 [0.23–0.42]	**<0.001**	0.37 [0.26–0.51]	**< .001**
Semantic memory and lang.	0.63 [0.50–0.80]	**<0.001**	0.88 [0.63–1.24]	0.479
Executive functions	0.54 [0.41–0.68]	**<0.001**	0.51 [0.35–0.72]	**<0.001**
Visuospatial skills	0.69 [0.55–0.85]	**<0.001**	0.97 [0.72–1.30]	0.832

OR, odds ratio; CI, confidence interval. All predictors were entered as standardized values (z-scores for demographics/clinical scales; w-scores for cognitive composites); thus, ORs reflect the effect size per one standard deviation change. Statistically significant values (*p* < 0.05) are indicated in bold.

**FIGURE 3:**
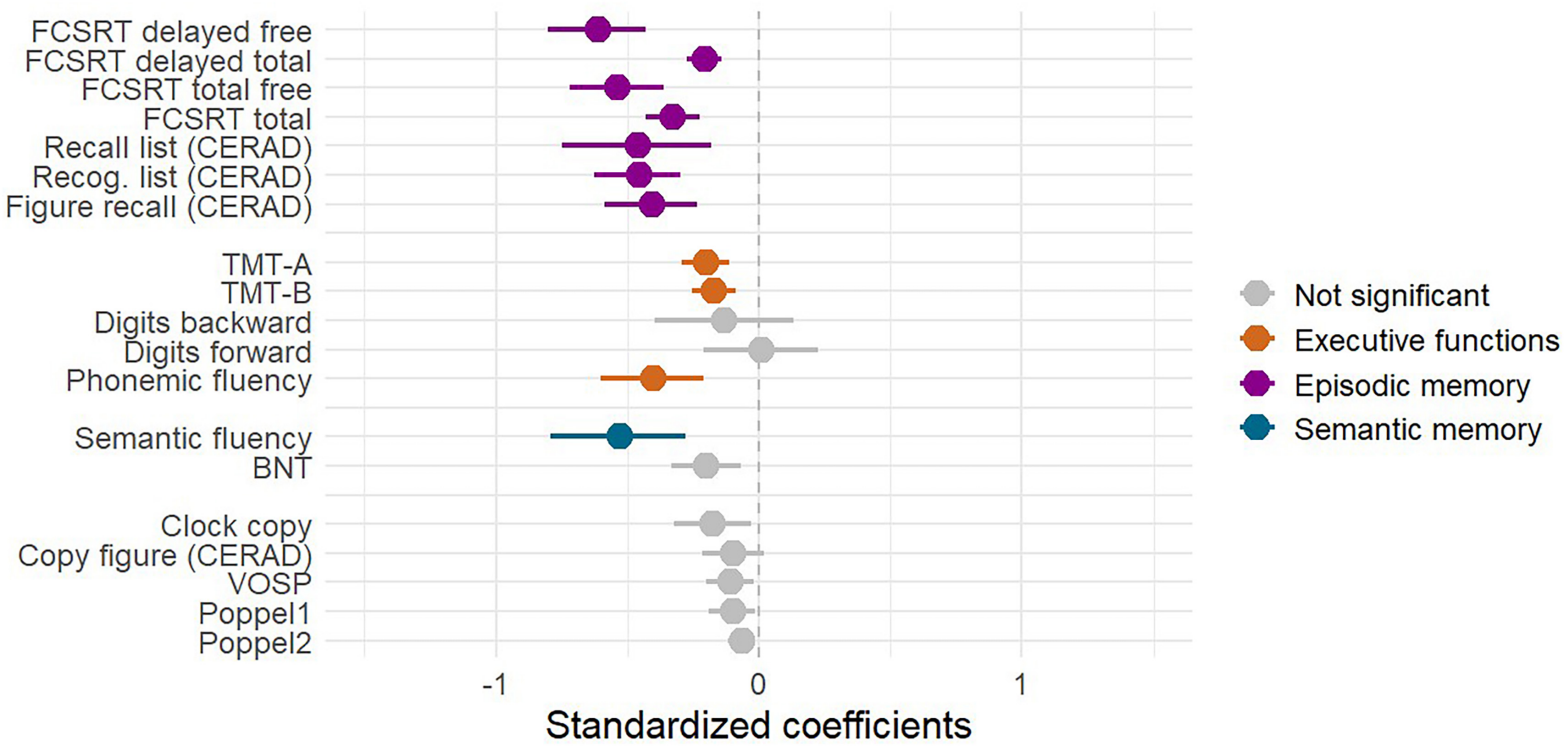
Logistic regressions to predict progression from MCI to dementia for each cognitive score. The circles represent the standardized coefficients for univariable models, and the lines represent the 95% confidence interval. *P*-values were corrected using the Bonferroni method for multiple comparisons (α = 0.05, *p* < 0.0026, 19 models considered). Gray color indicates non-significant values, while other colors indicate significant values. FCSRT, Free and Cued Selective Reminding Test; recog, recognition; CERAD the Consortium to Establish a Registry for Alzheimer’s Disease; BNT, Boston Naming Test; TMT-A and TMT-B, Trail-Making A and B; VOSP, Number Location condition from the Visual Object Spatial Perception; Poppel, Poppelreuter test.

Additional sensitivity analyses considering each test separately revealed that episodic memory measures provided the highest prognostic value. Specifically, the FCSRT delayed free recall (OR = 0.54, 95% CI: 0.45–0.65) was associated with a 46% reduction in conversion risk for every 1 SD increase in score. Significant protective effects were also observed for semantic fluency (41% risk reduction; OR = 0.59, 95% CI: 0.45–0.76) and phonemic fluency (33% risk reduction; OR = 0.67, 95% CI: 0.55–0.81). In contrast, measures of attention and working memory, including Digit Span (forward and backward), visuospatial tests, such as Clock Copy, CERAD figure copy, VOSP, and Poppel tasks, failed to significantly predict conversion risk, with Odds Ratios close to 1.0 and 5% confidence intervals including 1 (Figure 3 and Table 3).

**TABLE 3:** Univariable logistic regression results for neuropsychological predictors of conversion.

Test	Odds ratio [95% CI]	*z*-value	*p*-value
Figure recall (CERAD)	0.67 [0.56–0.79]	–4.52	**<0.001**
FCSRT delayed free	0.54 [0.45–0.65]	–6.44	**<0.001**
FCSRT delayed total	0.81 [0.76–0.87]	–6.23	**<0.001**
FCSRT total free	0.58 [0.49–0.70]	–5.88	**<0.001**
FCSRT total	0.72 [0.65–0.80]	–6.26	**<0.001**
Recall list (CERAD)	0.63 [0.47–0.83]	–3.19	**0.001**
Recog. list (CERAD)	0.63 [0.53–0.74]	–5.48	**<0.001**
TMT-A	0.82 [0.74–0.89]	–4.29	**<0.001**
TMT-B	0.84 [0.78–0.92]	–4.03	**<0.001**
Digits forward	1.01 [0.81–1.25]	0.08	0.935
Digits backward	0.88 [0.67–1.14]	–0.98	0.325
Phonemic fluency	0.67 [0.55–0.81]	–4.04	**<0.001**
Semantic fluency	0.59 [0.45–0.76]	–4.04	**<0.001**
Boston Naming Test (BNT)	0.82 [0.72–0.93]	–2.97	0.003
Clock copy	0.84 [0.72–0.97]	–2.33	0.020
Figure copy (CERAD)	0.91 [0.81–1.02]	–1.62	0.105
VOSP	0.90 [0.82–0.98]	–2.36	0.018
Poppel 1	0.91 [0.83–0.99]	–2.26	0.024
Poppel 2	0.94 [0.89–0.99]	–2.43	0.015

OR, odds ratio; CI, confidence interval; FCSRT, Free and Cued Selective Reminding Test; TMT, Trail Making Test; CERAD, Consortium to Establish a Registry for Alzheimer’s Disease; VOSP, Visual Object and Space Perception Battery. All tests were standardized using w-scores. *P*-values surviving Bonferroni correction for multiple comparisons are highlighted in bold (α = 0.05, *p* < 0.0026, 19 models considered).

### Multivariable analysis: from standard inference to regularized prediction

To assess the relative contributions of the variables while controlling for confounders, we first implemented a standard (unpenalized) multivariable logistic regression as a preliminary inferential step (Figure 2 and Table 2). In this model, male sex emerged as the strongest independent risk factor, with men being 2.73 times more likely to progress than women (*p* < 0.001, OR = 2.73, 95% CI: 1.53–5.01). Advanced age and higher education were also identified as independent risk factors. Specifically, each SD increase in age was associated with a 70% increase in the odds of conversion (*p* < 0.01, OR = 1.70, 95% CI: 1.17–2.50), while each SD increase in education contributed to a 47% increase in progression risk (*p* < 0.05, OR = 1.47, 95% CI: 1.05–2.08). Among cognitive measures, only episodic memory (*p* < 0.001, OR = 0.37, 95% CI: 0.26–0.51) and executive functions (*p* < 0.001, OR = 0.51, 95% CI: 0.35–0.72) retained independent predictive value, associated with a 63 and 49% reduction in conversion risk, respectively. Notably, global screening (MMSE), semantic memory, and visuospatial skills did not contribute additional predictive power to the final model, suggesting their influence is largely accounted for by the primary cognitive predictors.

**FIGURE 4:**
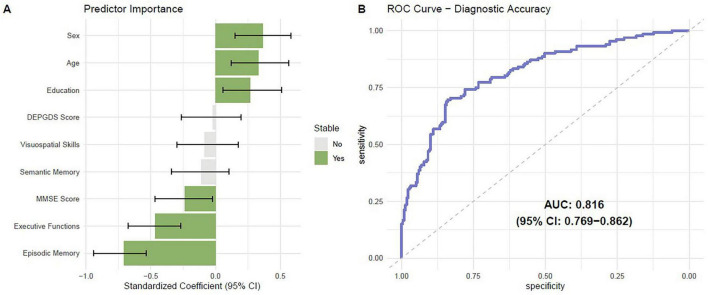
Predictor importance and ROC curve of the Ridge logistic regression model following a data-driven selection of α. **(A)** Standardized coefficients (β) and their stability (95% confidence intervals) based on 500 bootstrap iterations of the Ridge logistic regression model (α = 0, selected within an Elastic Net tuning grid); variables are considered stable predictors when the interval does not cross zero. **(B)** Receiver Operating Characteristic (ROC) curve representing the discriminatory power of the integrated model. CI, confidence interval; AUC, area under the curve.

However, to address the limitations of standard regression in the presence of multicollinearity, where highly correlated cognitive scores can lead to unstable estimates and inflated standard errors, we conducted a regularized Ridge logistic regression as our definitive predictive analysis. This model, optimized via an Elastic Net tuning grid (α = 0), provided a more stable assessment of predictor importance by employing coefficient shrinkage (Figure 4). The resulting set of predictors was further validated via bootstrap resampling, with the final model achieving an AUC of 0.816 (95% CI: 0.769–0.862). Episodic memory emerged as the most powerful individual predictor (β = -0.709), where a one-standard-deviation increase in baseline performance was associated with a 51% reduction in the odds of conversion. This was followed by executive functions (β = -0.466) and baseline MMSE scores (β = -0.237), which provided additional protective effects (risk reductions of 37 and 21%, respectively). Conversely, male sex (β = 0.366, 44% risk increase), older age (β = 0.331, 39% risk increase), and higher education (β = 0.268, 31% risk increase) were the primary drivers of clinical progression. At the optimal Youden threshold (0.436), the model’s good specificity (83.9%) and Negative Predictive Value (0.820) underscore its clinical utility as a screening tool to effectively rule out conversion in stable patients. Furthermore, the model demonstrated a sensitivity of 69.7%, indicating that it successfully identifies approximately 7 out of 10 progressors, balancing good diagnostic performance with practical clinical utility.

In contrast to the standard model, the Ridge framework identified the MMSE score as a stable predictor (β = -0.237, with bootstrap 95% CI excluding zero). This discrepancy in MMSE and education significance between the two models reflects their different statistical mechanics: while unpenalized estimation often fails to identify the independent contribution of global scales when specific domains (e.g., memory) are present, Ridge regression stabilizes these estimates by distributing weights among correlated features. Consequently, the Ridge model achieved a superior and more robust discriminatory power (AUC = 0.816, 95% CI: 0.769–0.862) compared to the unpenalized approach.

To evaluate the impact of follow-up duration on model performance, a subgroup analysis was conducted restricted to patients with a 3-year follow-up (*n* = 210). The model maintained good discriminatory power (AUC = 0.803, 95% CI [0.733–0.874]), indicating that the predictive value of the cognitive framework remains stable across the different follow-up intervals.

### Optimal predictive model

**FIGURE 5:**
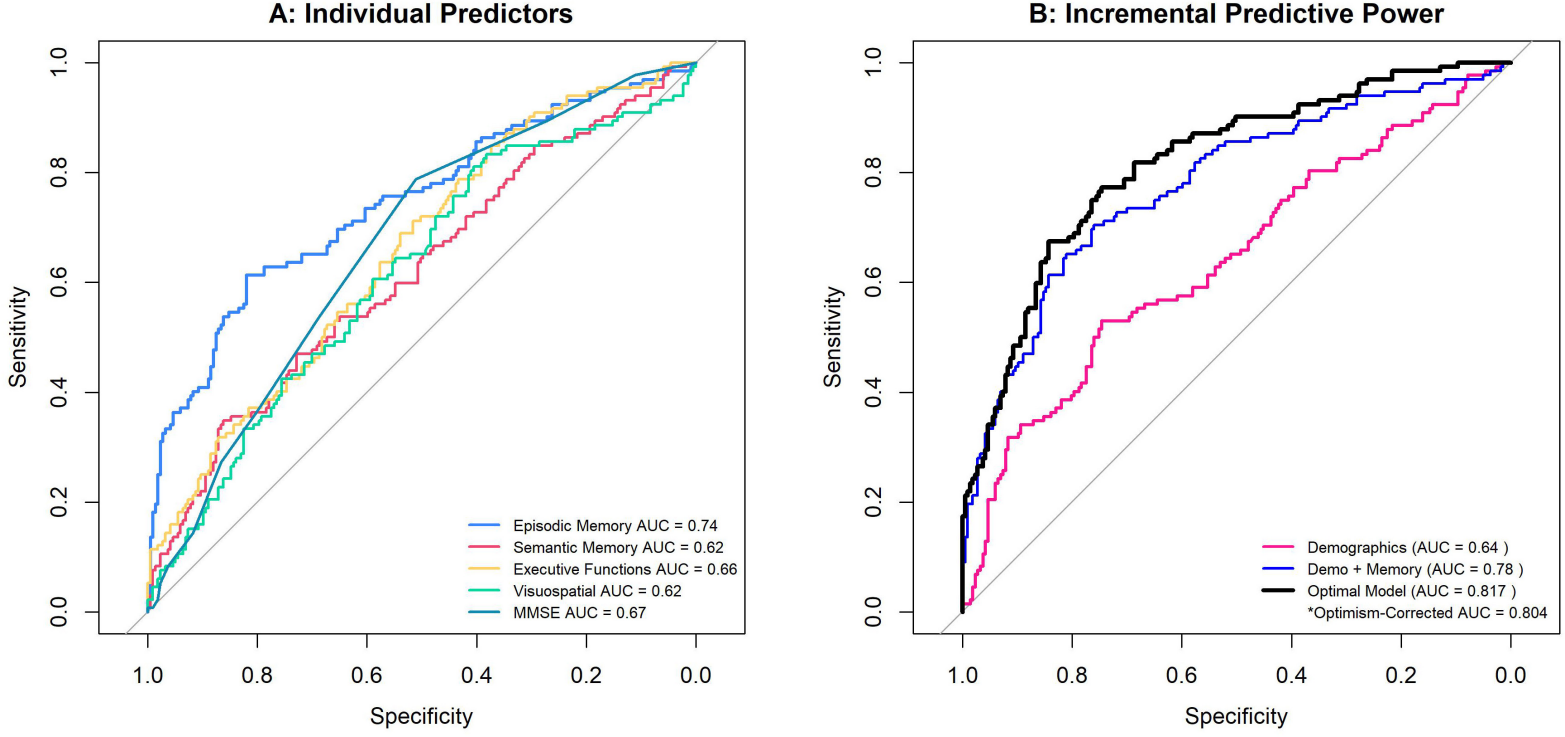
ROC curve analysis and predictive model comparisons. **(A)** Individual Predictors: Diagnostic performance of baseline cognitive measures, including episodic memory (W-score), MMSE, and other cognitive domains. **(B)** Incremental predictive power: comparison of clinical baselines with the integrated Ridge regression model (α = 0). The legend displays the apparent AUC for each model; for the final integrated model (black line; AUC = 0.816, 95% CI [0.769, 0.862]), the optimism-corrected AUC (derived from 500 bootstrap iterations) is also reported to reflect validated performance. To ensure consistency, all multivariable comparisons utilized the same regularized architecture (Ridge penalty, α = 0), while univariable predictors utilized standard logistic regression. All predictors were standardized across models to ensure that performance differences reflect the incremental predictive value of the included variables rather than variations in estimation algorithms or data scaling. MMSE, Mini-Mental State Examination; AUC, area under the curve; CI, confidence interval.

The Ridge logistic regression, following a data-driven selection of α (Figure 4), was selected as the optimal model. This model yielded a predictive profile encompassing episodic memory, executive functions, age, sex, education, and MMSE scores. The integrated Ridge model demonstrated good discriminatory power with an apparent AUC of 0.816 (95% CI: 0.769–0.862). After adjusting for model optimism through 500 bootstrap iterations, the optimism-corrected AUC remained robust at 0.804 (Figure 5).

We used Receiver Operating Characteristic (ROC) curves to assess the sensitivity and specificity of different variables and the optimal predictive model. To maintain methodological consistency, all comparison models were evaluated under a unified framework. Multivariable baseline models (e.g., demographics) were constructed using the same regularized regression architecture as the optimal model (Ridge penalty, α = 0). For univariable predictors, where regularization is mathematically equivalent to standard estimation for ROC analysis, logistic regression was employed. All predictors were standardized consistently across all models. This approach ensures that performance differences are attributable solely to the incremental predictive value of the included variables rather than variations in the estimation algorithm or data scaling (Figure 5).

Pairwise comparisons of the ROC curves using the DeLong test confirmed that the integrated Ridge optimal model significantly outperformed all individual cognitive predictors (Figure 5), including episodic memory (*Z* = 3.819, *p* < 0.001) and MMSE scores (*Z* = 5.140, *p* < 0.001). Furthermore, the integrated model showed significantly better discriminatory power than the demographics-only model (*Z* = 5.490, *p* < 0.001) and the combined demographics and episodic memory model (*Z* = 2.555, *p* = 0.011). Detailed test statistics for all comparisons are provided in Supplementary Table 3.

## Discussion

We explored the predictive value of neuropsychological measures and demographic variables in the 3-year evolution of individuals with MCI and developed a predictive model to forecast the progression from MCI to dementia. In our cohort, 132/349 (37.8%) MCI patients converted to dementia after an average of 3.1 years (ranging from 2.6 to 3.4 years) of follow-up. The converters were significantly older and performed worse at baseline than non-converters (stable and reverters groups) in all cognitive measures. A regularized (Ridge) logistic regression, selected as our definitive analysis through an Elastic Net tuning grid, revealed that the optimal model to predict conversion to dementia includes Episodic memory, Executive functions, MMSE scores, Sex, Education, and Age. The stability of these predictors was confirmed via 500 bootstrap iterations, yielding a good performance (AUC = 0.816, 95% CI: 0.769–0.862) to identify individuals who converted to dementia. This underscores the clinical relevance of integrating demographic data with specific cognitive profiles. Our model’s AUC of 0.816 outperforms standard bedside tools, such as the MMSE (AUC ≈ 0.68), the ADAS-Cog (AUC ≈ 0.67), while remaining highly competitive with specialized instruments like CAMCOG memory section (AUC ≈ 0.80), ACE (AUC ≈ 0.98), or the MoCA (Lischka et al., 2012). The study found that while traditional logistic regression failed to identify MMSE and education as significant (due to multicollinearity), the Ridge model successfully captured their predictive value. By using coefficient shrinkage to distribute weights among correlated variables rather than masking them, Ridge regression revealed that global cognitive status (MMSE) and education are actually stable, independent predictors of dementia conversion.

Notably, this regularized regression framework outperformed individual predictors, such as the MMSE (AUC = 0.72), or Episodic memory in isolation (AUC = 0.77), as confirmed by DeLong test comparisons. This incremental gain suggests that, although episodic memory impairment remains a hallmark of progression, jointly modeling age, sex, education, MMSE, and executive function yields more stable 3-year risk estimates. From a clinical perspective, the model’s moderately high specificity (83.9%) and Negative Predictive Value (82.0%) support its utility in identifying patients unlikely to convert within 3 years. However, the moderate sensitivity (69.7%) implies that a non-trivial fraction of progressors would be missed; thus, continued longitudinal monitoring remains warranted. By relying on routinely available variables, the model offers a pragmatic option for specialized memory clinics, to be used alongside clinical judgment.

These findings have implications for clinical contexts with limited access to fluid or neuroimaging biomarkers. Specifically, the study identifies sensitive neuropsychological measures that can be prioritized for inclusion in a standard clinical visit. This is crucial given the typical time and economic constraints that preclude the application of a more exhaustive neuropsychological battery. By focusing the initial assessment on the most informative measures, clinicians gain the efficiency needed to potentially incorporate additional, more dementia-type-specific neuropsychological tests that would otherwise be impossible to administer within the limited consultation time.

In this study of 349 MCI patients at baseline, 132 (37.8%) converted to dementia, 205 (58.7%) remained stable, and 12 (3.4%) were reverters after an average of 3.1 years of follow-up. A significant proportion of converters developed AD dementia (41%). These numbers are consistent with other studies: for example, Tschanz et al. (2006) reported that approximately 46% of individuals with MCI develop dementia, with AD being the most likely form; Ganguli et al. (2019) observed that 60% of participants remained cognitively stable; and Petersen et al. (2018) found that from 14.4 to 38% of MCI returned to normal cognition. The fact that a majority of MCI patients remained stable after 3 years of follow-up does not rule out future conversion to dementia. The apparent stability may reflect an underlying neurodegenerative process that has not yet progressed enough to cause significant cognitive and functional decline.

Regarding demographic variables, age emerged as a significant predictor, with older MCI individuals more likely to convert to dementia. This finding is consistent with previous research showing that older individuals with MCI are at a higher risk of developing dementia compared to younger individuals (Petersen, 2016). Additionally, younger age has been associated with a higher likelihood of reverting to normal cognition in some studies (Cooper et al., 2015; Roberts and Knopman, 2013). The deleterious effect of age on MCI conversion may be explained by the increased prevalence of degenerative disease and pathologies with age (Chen et al., 2022; Boyle et al., 2021), and also the greater number of comorbidities (Petersen et al., 2014; Savva et al., 2009; Schneider et al., 2009.

In our study, sex was found to have a significant effect when it was included in the multivariable models, but not in the univariable model. In our multivariable models, male sex was associated with a higher risk of conversion, a finding that stands in contrast to some existing literature. The reasons for this discrepancy remain unclear. One possibility is that unmeasured variables, such as APOE ε4 status and pathophysiological biomarkers (e.g., amyloid PET, CSF, or plasma), may interact with sex and influence the risk of progression to dementia. Alternatively, this finding may reflect clinical selection effects. For instance, sex differences in healthcare-seeking behavior may result in men being referred to specialized memory clinics at a later stage of cognitive decline compared to women. Consequently, men in our cohort may have entered the study with more advanced, albeit sub-clinical, pathology, which could contribute to a faster observed conversion rate. Finally, we cannot exclude that these results also reflect characteristics specific to our local population.

Similarly, education did not have a clear effect on the prediction of MCI conversion. In the multivariable models, higher education emerged as a predictor of faster conversion, despite showing no significant univariate association. This kind of change can arise in several ways. One explanation is suppression: education may share irrelevant variance with other predictors (e.g., global cognitive performance), and once that is controlled for, its independent effect, possibly related to cognitive reserve, emerges. In this view, more educated individuals may tolerate greater neuropathology before meeting the clinical threshold for MCI, so they are diagnosed later in the disease process and then decline more quickly. Another possibility is collider bias, if the model controls for variables (e.g., MMSE) influenced by both education and neurodegeneration, thereby creating a spurious relationship. Because the study lacked biomarker data, such as amyloid-β, tau, or cerebrovascular measures, these explanations cannot be distinguished conclusively; future research should incorporate biomarkers and explicit causal models to clarify how education, cognitive reserve, confounding, and disease progression interact in MCI conversion.

Concerning cognitive variables, episodic memory, and executive functions were the strongest predictors of dementia progression. Interestingly, our findings revealed that semantic memory, language, and visuospatial skills did not significantly enhance the predictive performance of the final model. Our model showed that, separately, episodic memory has an AUC of 0.74 in predicting dementia conversion. This discriminatory power increased to an AUC of 0.816 when episodic memory was combined with executive functions, MMSE scores, and demographic variables. These findings align with other studies indicating that impairment in memory and other cognitive domains significantly increases the risk of AD (García-Herranz et al., 2016; Tabert et al., 2006). While episodic memory deficits are a hallmark of AD, recent research also highlights working memory, attention and executive functions deficits in the preclinical stage of AD (Ho and Nation, 2018; Kirova et al., 2015).

Finally, the predictive value of each cognitive test individually was assessed with a sensitivity analysis. Consistent with the main findings, episodic memory tests, especially the FCSRT delayed free recall, emerged as the most robust markers. These results are in line with previous research that underscores the strong FCSRT’s reliability as a predictor of conversion to dementia (Ewers et al., 2012; Grande et al., 2018). The non-episodic tests showing the highest predictive values consisted of time-based tests, including semantic and phonemic fluencies and the Trail Making Test. Previous research has documented the effectiveness of time-based measures in detecting early cognitive decline (Phillips et al., 2013). These tests offer several key advantages: they provide objective and continuous measurements, demonstrate greater score variability, avoid ceiling and floor effects common in other assessments, and effectively capture subtle changes in processing speed, and executive control that often characterize the earliest stages of cognitive deterioration (García-Herranz et al., 2016; Kirova et al., 2015; Tse et al., 2010).

This study has several strengths, including its large and well-characterized clinical cohort of MCI patients and the use of a comprehensive neuropsychological battery at baseline, which allowed for a thorough evaluation of cognitive functioning across multiple domains. We used an original approach (W-scores) to adjust for demographic factors, which may have, in turn, enhanced our predictive performance. However, some limitations should be acknowledged: (A) we did not use an independent sample to validate the predictive model; (B) some exclusion criteria limited the exploration of certain risk factors, such as depression; (C) the relatively small proportion of reverters in our sample (*n* = 12) may represent a methodological constrain for robust three-way group comparisons and should be interpreted as exploratory; (D) the inclusion of individuals with various types of dementia, in different proportions, may have influenced some results; (F) the absence of pathophysiological confirmation of the neurodegenerative diagnosis; (G) the use of a binary outcome to capture the critical clinical threshold (GDS ≥ 4) for functional dementia, as this approach aligns with real-word diagnostic processes; and finally, H) the prioritization of a parsimonious model to avoid overfitting and maintain statistical stability (Burnham and Anderson, 2002), despite the theoretical relevance of interactions between demographic variables and cognition.

It is worth mentioning that our aim was to predict dementia in a heterogeneous clinical cohort of MCI patients, using only cognitive and demographic measures rather than biomarker data. These findings hold significant practical implications for clinical neuropsychologists, working on clinical settings outside specialized tertiary centers, where biomarker testing and advanced neuroimaging are often not readily available. Our results are important as they demonstrate that a neuropsychological evaluation of less than an hour, together with basic demographic data, has good discriminatory power in predicting conversion to dementia in MCI patients from a clinical cohort.

While we acknowledge that a 1-h battery requires specialized training and represents a time commitment in busy clinical settings, these data offer a rationale for optimizing test selection. By prioritizing episodic memory and executive functions assessments over more traditional but less predictive measures (such as digit span), clinicians can maximize the diagnostic utility of a single session. This is especially relevant for resource-limited or rural settings; while these environments still face barriers regarding standardized materials and trained staff, a refined cognitive battery may represent a more accessible alternative to expensive biological markers.

Ultimately, utilizing these high-value diagnostic instruments allows for better risk stratification. Identifying at-risk patients early enables health professionals to provide proactive support, including education on risk-reduction measures, such as healthy diets, regular exercise, cognitive activities, and cardiovascular prevention, that may help delay the onset of neurodegenerative disease (Dubois et al., 2024; Petersen et al., 2018; Roberts and Knopman, 2013; Winblad et al., 2004).

## Conclusion

In this research, we assessed the predictive value of baseline neuropsychological assessments and demographic factors for identifying progression from MCI to dementia over a 3-year period. Within our cohort, 132 out of 349 MCI patients (37.8%) transitioned to dementia.

A regularized (Ridge) logistic regression, following a data-driven selection of α within an Elastic Net tuning grid, identified an optimal predictive model consisting of age, sex, education, MMSE, episodic memory, and executive functions. The stability of these predictors was confirmed through 500 bootstrap iterations, ensuring the reliability of the model’s weights in the absence of intrinsic sparsity. This framework demonstrated good discriminatory power (AUC = 0.816), outperforming individual demographic or cognitive variables alone.

These findings indicate that considering multiple cognitive domains along with basic demographic information provides good predictive value for the clinical trajectory of MCI over 3 years. This has significant implications for experienced neuropsychologists working at clinical settings with limited access to fluid or neuroimaging biomarkers. This information will enable them and their families to prepare for upcoming care requirements.

## Data Availability

The raw data supporting the conclusions of this article will be made available by the authors upon reasonable request.
