# Supplementary material for: The predictive power of neuropsychological measures in MCI: early detection of dementia conversion
Source: Front Aging Neurosci. 2026 Jun 18;18:1740033. doi: 10.3389/fnagi.2026.1740033 (PMC13329795; doi:10.3389/fnagi.2026.1740033)
Supplement: Supplementary file 1 [file Table_1.pdf]

Supplementary Table 1. Missing Values by Neuropsychological Subtest and Clinical Group

**Variable (w scores)**	**Total Missing Values (%)**	**Converter (n=132)**	**Stable (n=205)**	**Reverter (n=12)**
Rey Osterrieth Complex Figure Test Delayed Recall	202 (57.9%)	84 (63.6%)	113 (55.1%)	5 (41.7%)
CERAD Constructional Praxis Delayed Recall	13 (3.7%)	7 (5.3%)	6 (2.9%)	0 (0.0%)
FCSRT Delayed Free Recall	6 (1.7%)	2 (1.5%)	4 (2.0%)	0 (0.0%)
FCSRT Delayed Total Recall	6 (1.7%)	2 (1.5%)	4 (2.0%)	0 (0.0%)
FCSRT Total Free Recall	6 (1.7%)	2 (1.5%)	4 (2.0%)	0 (0.0%)
FCSRT Total Recall	6 (1.7%)	2 (1.5%)	4 (2.0%)	0 (0.0%)
CERAD Word List Delayed Recall	48 (13.8%)	30 (22.7%)	18 (8.8%)	0 (0.0%)
CERAD Word List Recognition	48 (13.8%)	30 (22.7%)	18 (8.8%)	0 (0.0%)
Semantic Verbal Fluency (Animals)	2 (0.6%)	1 (0.8%)	1 (0.5%)	0 (0.0%)
Boston Naming Test	5 (1.4%)	0 (0.0%)	5 (2.4%)	0 (0.0%)
Phonemic Verbal Fluency (Letters)	2 (0.6%)	1 (0.8%)	1 (0.5%)	0 (0.0%)
Trail Making Test Part A	3 (0.9%)	0 (0.0%)	3 (1.5%)	0 (0.0%)
Trail Making Test Part B	60 (17.2%)	28 (21.2%)	32 (15.6%)	0 (0.0%)
Rey Osterrieth Complex Figure Test Execution Time	205 (58.7%)	87 (65.9%)	114 (55.6%)	4 (33.3%)
Digit Span Forward	8 (2.3%)	3 (2.3%)	5 (2.4%)	0 (0.0%)
Digit Span Backward	8 (2.3%)	3 (2.3%)	5 (2.4%)	0 (0.0%)
Comprehension subtest Assessment Scale-Cognition (COMPADAS-15)	261 (74.8%)	95 (72.0%)	154 (75.1%)	12 (100.0%)
Rey Osterrieth Complex Figure Test Copy	196 (56.2%)	82 (62.1%)	110 (53.7%)	4 (33.3%)
Clock Drawing Test Command	6 (1.7%)	2 (1.5%)	4 (2.0%)	0 (0.0%)
CERAD Constructional Praxis Copy	13 (3.7%)	7 (5.3%)	6 (2.9%)	0 (0.0%)
Visual Object and Space Perception Battery (VOSP)	7 (2.0%)	3 (2.3%)	4 (2.0%)	0 (0.0%)
Poppelreuter Test Part 1	3 (0.9%)	2 (1.5%)	1 (0.5%)	0 (0.0%)
Poppelreuter Test Part 2	3 (0.9%)	2 (1.5%)	1 (0.5%)	0 (0.0%)

Percentage of missing values for each neuropsychological subtest across the entire Mild Cognitive Impairment cohort and stratified by clinical group. Note: CERAD = Consortium to Establish a Registry for Alzheimer's Disease; FCSRT = Free and Cued Selective Reminding Test.
