# Supplementary material for: The predictive power of neuropsychological measures in MCI: early detection of dementia conversion
Source: Front Aging Neurosci. 2026 Jun 18;18:1740033. doi: 10.3389/fnagi.2026.1740033 (PMC13329795; doi:10.3389/fnagi.2026.1740033)
Supplement: Supplementary file 2 [file Table_2.pdf]

Table S2. Clinical Progression Summary

Clinical Group	Final GDS Score	Count (n)	Percentage (%)
Converters (*n*=132)	4	115	87.1%
	5	11	8.3%
	6	5	3.8%
	7	1	0.8%
Reverters (*n*=12)	1	1	8.3%
	2	11	91.7%
Stable (*n*=204)	3	205	100.0%
Total (N=349)		349	100%
