# Supplementary material for: The predictive power of neuropsychological measures in MCI: early detection of dementia conversion
Source: Front Aging Neurosci. 2026 Jun 18;18:1740033. doi: 10.3389/fnagi.2026.1740033 (PMC13329795; doi:10.3389/fnagi.2026.1740033)
Supplement: Supplementary file 3 [file Table_3.pdf]

Table S3. Statistical Comparison of ROC Curves (DeLong Test)

Model Comparison	Z-Statistic	p-value
Optimal Model vs. Episodic Memory	3.819	< 0.001
Optimal Model vs. Semantic Memory	6.093	< 0.001
Optimal Model vs. Executive Function	5.439	< 0.001
Optimal Model vs. Visuospatial	5.809	< 0.001
Optimal Model vs. MMSE	5.140	< 0.001
Optimal Model vs. Demographics	5.490	< 0.001
Optimal Model vs. Demo + Memory	2.555	< 0.05
