# Supplementary material for: The predictive power of neuropsychological measures in MCI: early detection of dementia conversion
Source: Front Aging Neurosci. 2026 Jun 18;18:1740033. doi: 10.3389/fnagi.2026.1740033 (PMC13329795; doi:10.3389/fnagi.2026.1740033)
Supplement: Supplementary file 4 [file Image_1.pdf]

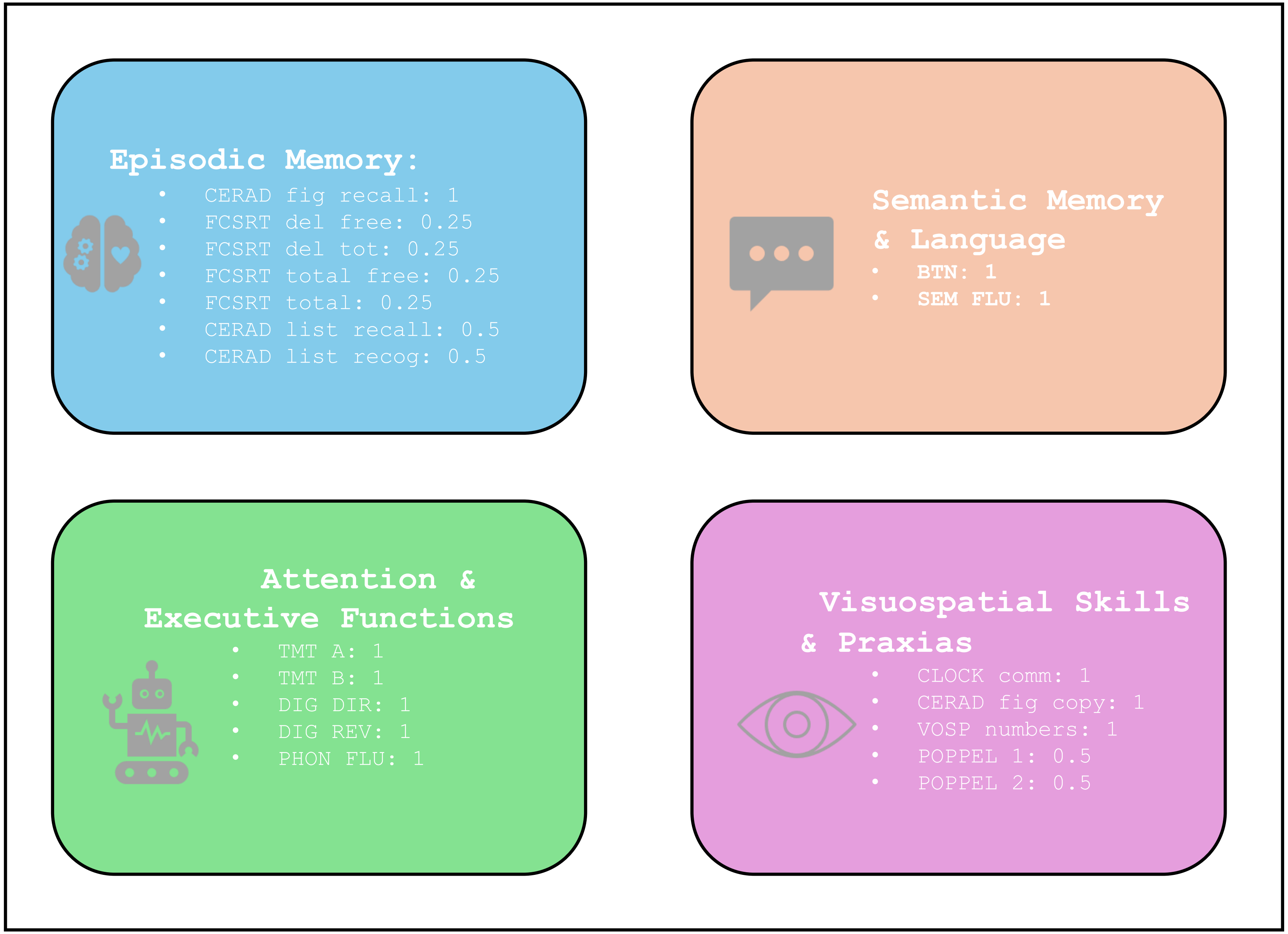
Episodic Memory:
CERAD fig recall: 1
FCSRT del free: 0.25
FCSRT del tot: 0.25
FCSRT total free: 0.25
FCSRT total: 0.25
CERAD list recall: 0.5
CERAD list recog: 0.5
Semantic Memory & Language
BTN: 1
SEM FLU: 1
Attention & Executive Functions
TMT A: 1
TMT B: 1
DIG DIR: 1
DIG REV: 1
PHON FLU: 1
Visuospatial Skills & Praxias
CLOCK comm: 1
CERAD fig copy: 1
VOSP numbers: 1
POPPEL 1: 0.5
POPPEL 2: 0.5
